# A19 PROTEOMICS FOR PREDICTING NECROTIZING ENTEROCOLITIS IN THE PREMATURE NEONATE

**DOI:** 10.1093/jcag/gwab049.018

**Published:** 2022-02-21

**Authors:** E Shajari, D Gagné, M Thibault, É Tremblay, D Gryspan, E Ferretti, V Bertelle, J Beaulieu

**Affiliations:** 1 Universite de Sherbrooke Faculte de Medecine et des Sciences de la Sante, Sherbrooke, QC, Canada; 2 University of Ottawa Faculty of Medicine, Ottawa, ON, Canada; 3 The University of British Columbia Faculty of Medicine, Vancouver, BC, Canada

## Abstract

**Background:**

Necrotizing enterocolitis (NEC) represents a major challenge in neonatal intensive care units (NICU). The search for indicators that could be used to predict the development of NEC, which would provide more time to apply targeted interventions in the NICU before the appearance of the symptoms is required.

**Aims:**

The aim of the present work was to investigate the potential of fecal proteomics signatures for NEC prediction

**Methods:**

In the present study, stools from 132 very low birth weight infants (less than 1500 g and born younger than 30 weeks) were collected daily in the context of a multi-center prospective study. Seven of the infants received a stage 3 NEC diagnosis. Stools collected up to 10 days before diagnosis were included and each NEC was matched with 2 non-NEC controls. These samples had been used to evaluate various biomarkers by ELISA in a previous study, which revealed that lipocalin-2 and calprotectin used in conjunction can allow the prediction of half of very low birth weight infants 7 days before their NEC diagnosis (Thibault et al., Ped Res 2021). Herein, we explore a proteomics approach to investigate whether this predictability can be improved. The same stool samples were thus prepared and processed for liquid chromatography-tandem mass spectrometer analysis (TripleTOF 5600) coupled with SWATH acquisition software.

**Results:**

Data were analyzed by Skyline using the peptide transition list of a spectral library leading to the identification of 1374 proteins with a minimum of two peptides. From these, 192 proteins (1061 peptides) were detected at strong levels in a majority of the samples while 37 of them (2–4 peptides/protein, 102 peptides) were found to display significantly altered levels between NEC and non-NEC samples (17 up; 20 down) based on statistical analyzes and displaying an AUC ≥ 0.7 (ROC curve). Interestingly, both sets of peptides for the NEC samples were significantly different from controls for all three tested periods (group 1: -10 to -7, group 2: -6 to -3, and group 3: -2 to +1 days before diagnosis) using One-way ANOVA Dunnett’s multiple comparison test, p < 0.001. Furthermore, analyzing the data for each infant confirmed the usefulness of the peptide signature for predicting NEC development in 6 of the 7 available cases one week in advance of the diagnosis.

**Conclusions:**

Taken together, these results indicate that stool proteomics represents a promising potential approach for predicting NEC in very low-weight infants.

**Funding Agencies:**

CIHR

